# The validity of routine individuated programmatic data in HIV surveillance assessed over a 10-year period.

**DOI:** 10.23889/ijpds.v7i3.1840

**Published:** 2022-08-25

**Authors:** Nisha Jacob, Brian Rice, Andrew Boulle

**Affiliations:** 1 University of Cape Town; 2 London School of Hygiene and Tropical Medicine

## Objectives

In the Western Cape, South Africa, public-sector individual-level routine data are consolidated from multiple non-EMR sources through the Provincial Health Data Centre (PHDC). This enables the description of temporal changes in population-wide antenatal HIV sero-prevalence. We evaluated the validity of these data compared to aggregated program data and sentinel surveys.

## Approach

We conducted a retrospective cohort analysis of all pregnancies consolidated in the PHDC from January 2011 to December 2020. Evidence of antenatal and HIV care from electronic platforms (each with its own multi-source phenotype algorithm) were linked using a unique patient identifier. HIV prevalence estimates were triangulated with available sentinel national and provincial antenatal survey estimates as well as aggregated programmatic data from registers as recorded in the District Health Information System. Provincial, district-level and age-group HIV prevalence estimates were compared between data systems using correlation coefficients, absolute differences and trend analysis.

## Results

In total, 982,914 pregnancies were ascertained with a median maternal age of 26.9 years. Between 2011–2013, PHDC HIV prevalence estimates were widely disparate from aggregate and survey data (due to incomplete electronic data) whereas, from 2014 onwards, estimates were more closely correlated to aggregate data estimates (r=0.83; p=0.02) with an average absolute prevalence difference of 0.97%. In keeping with survey and aggregate data trends, PHDC data show a relatively stable provincial HIV prevalence from 17.0 (95%CI 16.8%–17.2%) in 2015 to 18.9% (95%CI 18.7–19.1%) in 2020. The highest HIV prevalence was in the Cape Metro district (20.6%; 95%CI 20.4%–20.9%). Prevalence estimates by age group were comparable between sentinel surveys and PHDC from 2014 onwards with an average absolute prevalence difference of 1.6%.

## Conclusion

This study is the first to compare sentinel sero-prevalence surveys with both register-based aggregate data and consolidated individuated administrative data. We show that in this setting linked individuated data may be reliably used for HIV surveillance and provide more granular estimates with greater efficiency.

